# Massachusetts Veterinary Association

**Published:** 1893-12

**Authors:** 


### SOCIETY PROCEEDINGS.

### MASSACHUSETTS VETERINARY ASSOCIATION.

The regular meeting of the Massachusetts Veterinary Association was held at 19 Boylston Place, Boston, Wednesday evening, January 25th, 1893, at 7.30 o'clock.

President L. H. Howard in the chair. Members present, Drs. Winchester, Osgood, Winslow, Stinson, La Baw, Blackwood, Marshall, Hadcock, Bryden, Parker, Emerson, Beckett, Burr and Harrington. Honorary member, Dr. Stickney. Visitor, Mr. Naylor. In the absence of the secretary, the President appointed Dr. Parker secretary *pro tem.* Unfinished business, none.

*Reports of Committees.—Dr. Osgood* for the Executive Committee reports favorably upon the credentials of H. P. Rogers, M.D.V. (Harv.).

*Dr. Winchester* reports a meeting of the U. S. V. M. A. comitia minora in New York, January 20th, when it was decided to hold the next annual meeting in October. instead of September. This was necessary on account of several different congresses meeting in Chicago in September.

Admission of members.—Names of C. R. Simpson, D.V.S., and T. W. Shaw, D. V. S., were balloted upon. Eleven votes cast, all in the affirmative ; they were therefore declared elected.

*Dr. La Baw* then read a paper upon "Punctured Wounds of the Foot." A general discussion of the question followed, taken part in by most of the members present, the debate not only involving punctured wounds of the foot, but its sequelæ such as quittors, synovitis, ringbone and tetanus. Motion made by Dr. Winchester, seconded by Dr. Winslow, that the essayist be given a vote of thanks. Carried.

*Dr Osgood* then introduced an agent from a Chicago firm of instrument makers, who invited the members of the Association to inspect samples of veterinary instruments at the Harvard Veterinary School. After a desultory discussion on the respective merits of hot and cold applications in synovitis and laminitis ; toe and quarter cracks ; and spavin, its causes and treatment, the meeting adjourned.

J. M. PARKER, Secretary *pro tempore.*

---

The regular meeting of the Massachusetts Veterinary Association was held at 19 Boylston Place, Wednesday evening, February 22nd. 1893.

President L. H. Howard in the chair. Members present, Drs. Howard, Marshall, Peterson, Winchester, Winslow, Parker, and the Secretary. Honorary member, Dr. Stickney.

Records of the last two meetings read and accepted. Unfinished business, none. Reports of committees, none.

Admission of members.—Name of Howard P. Rogers, M. D. V. (Harv.), balloted upon ; seven votes cast, six in the affirmative and one blank ; the President declares Dr. Rogers elected.

Papers and Discussions.—Dr. Blackwood was to have read a paper, but he was absent, so no paper was read.

*Dr. Parker* reported a case of azoturea ; it was like any case of this disease, with the exception that it came on without any exercise to speak of to induce it ; the animal had been idle in the stable for a week, and dropped down with azoturea in walking back from the water-trough to its stall, it having just taken a drink of water.

The members present then discussed the humbuggery of the Massachusetts law providing for the appointment of a cattle inspector in every city and town in the State by the Mayor or Selectmen. In places where competent men were appointed the law works well enough : for example, Dr. Burr in Boston, and Dr. Peterson in Waltham and Watertown. On the other hand the cattle inspector in Wayland is a painter, in Andover a blacksmith, in North Andover a farmer, and so on through a long list that makes an attempt at the suppression of tuberculosis or other infectious cattle diseases little better than a farce. A discussion of meat inspection in abattoirs followed, considering the danger, if any, from actinomycosis, and also the question where is the line to be drawn between a tuberculosis carcass fit for use as human food, and one so bad that it must be thrown away ?

Meeting then adjourned. AUSTIN PETERS, Secretary.

---

The regular meeting of the Massachusetts Veterinary Association was held at 19 Boylston Place, Wednesday evening, March 22nd, 1893, at seven-thirty o'clock.

President L. H. Howard in the chair. Members present, Drs. Bryden, Blackwood, Burr, Emerson, Hadcock, Howard, Winchester, Winslow, Parker, La Baw, Carlton, Simpson, Rogers and the Secretary. Honorary member, Dr. Stickney.

Minutes of the previous meeting read and accepted.

Applications for membership.—Application of Dr. E. H. Holden, of Springfield, received and laid on the table for a month under the rules.

The President calls the attention of the members to the fact that it is time to take action upon our annual meeting. Dr. Winchester makes the motion : "That the annual meeting and dinner be held at Young's Hotel, and that a committee of three be appointed by the chair to arrange the details, and that the President be one of the committee." Seconded by Dr. Parker. Motion put by the Secretary and carried unanimously.

*The Chair* announces Drs. Winchester and Osgood as the other two members of the Committee of Arrangements.

*Dr. Winchester* moves : "That the hour of the annual meeting be five-thirty." Seconded by Dr. Winslow. Carried.

*Dr. Winslow* moves : "That the cost of the dinner be restricted to $5.00 per plate" Not seconded.

*Dr. Parker* moves : "That the price of the dinner be left to the discretion of the Committee." Seconded by Dr. Simpson. Carried.

*Dr. Winslow* moves : "That the guests to be invited be left to the discretion of the Committee." Seconded by Dr. Simpson. Carried.

The Secretary informed the members that Dr. Liautard had just sent them a copy of his new work on surgery, for the Association's library, and also a lot of back numbers of the Review, which now makes the file of the Association complete, with the exception of two numbers missing, in different years, which are now out of print. Moved by Dr. Winchester, that the Association tender Dr. Liantard a vote of thanks for his contributions to its library. Seconded by Dr. Blackwood. Carried.

Moved by Dr. Winchester: "That the Association's vote of thanks be given to the Secretary for some odd numbers of Fleming's Journal, which he had given to make the file complete." Seconded by Dr. Blackwood. Carried.

*Dr. Parker* read a very interesting paper on "Scouring in Calves," * followed by a general discussion, in which the members present took part.

*Dr. Winchester* remarked that he thought the paper one of the best ever read before the Association, and moved that the essayist be given a vote of thanks for his paper. Seconded by Dr. Stickney. Carried.

*Dr Parker* then reported a case and showed a specimen of, thrombosis of the external iliac artery. The clot in the external iliac was several inches long and completely filled the lumen of the vessel; there were also two parasitic aneurisms in the coeliac. The thrombus could not be felt ante-mortem per rectum, as the plug was too low down, but the leg was cold and the horse was lame on it (off hind), there was inflammation of the laminæ, separation at hair and hoof, periostitis of os-pedis, and caries of part of it, colicky pains at times, at other times would have dizzy spells and throw himself down and thrash around. Was finally shot.

Meeting then adjourned. AUSTIN PETERS, Secretary.

---

The tenth annual meeting of the Massachusetts Veterinary Association was held at Young's Hotel, Boston, on Wednesday evening, April 26th, 1893.

Dr. L. H. Howard in the chair. There were present Drs. Bryden, Blackwood, Howard, Osgood, Saunders, Winchester, Winslow, Marshall, Burr, Beckett, Simpson and Rogers. Honorary members, Drs. Liautard and Stickney.

The minutes of the previous meeting having been read. Dr Bryden desired to make a correction. At the previous meeting he had called attention to Dr. Bolton's report on the case of the spring-halt horse; that report he thought would convey the impression that the horse was killed in consequence of the failure of the treatment instituted by him, when in fact the horse was procured with intention and for the purpose of killing it for p. m. examination. Dr. Bryden further called attention to the fact that the patient distinctly improved while under treatment.

The minutes were then accepted with this correction.

Admission of members.—Dr. E. H. Holden was unanimously elected a member, ten votes being cast, all in the affirmative.

*Election of Office Bearers.*—For *President*, *Dr. Burr*, nominated by Dr. Osgood. seconded by Dr. Winslow, unanimously elected. Dr. Burr then took the chair. Dr. Bryden then nominated *Dr. Beckett* for *First Vice-President;* seconded by Dr. Winchester; unanimously elected. Dr. Howard nominated *Dr. Winslow for Second Vice-President;* seconded by Dr. Winchester; unanimously elected. *For Secretary and Treasurer*, *Dr. Parker;* moved by Dr. Osgood, seconded by Dr. Winchester; elected.

---

*See page 227, October Journal.

*Executive Committee:* *Drs. Blackwood, Osgood, Labaw and Saunders*, in order.

There being no new business the members adjourned to the banquet hall, where the annual dinner was partaken of, and where the genial toastmaster, Dr. Bryden, kept the ball rolling till a late hour.

JOHN M. PARKER, Secretary.

---

The regular meeting of the Massachusetts Veterinary Association was held a 19 Boylston Place, May 24th, 1893, at seven-thirty o'clock P. M.

There were present Drs. Blackwood, Howard, Osgood, Winchester, Winslow, Burr, Labaw, Simpson and Rogers. Honorary member, Dr. Stickney.

The President, Dr. Burr, in the chair.

The minutes of the last meeting having been read, a long discussion ensued as to whether that portion of the minutes which refers to the meeting previous to the annual meeting should be adopted. Some of the members were of the opinion that if any reference were made to the discussion relating to the "Spring-halt horse," the record of the discussion should be more complete.

*Dr. Blackwood* moved "That the minutes be accepted as read." Seconded by Dr. Winchester.

*Dr. Howard* then moved as amendment, "That the records, or such portions of the records of the Annual Meeting as refers to the meeting previous to the Annual Meeting, *i.e.* the March Meeting, be not accepted until such records of previous (March) meeting be reconsidered and corrected by Dr. Peters." Seconded by Dr. Osgood. Carried.

*New Business.—Dr. Osgood* then read a letter from Dr. Peters, enclosing on from Mr. Appleton, in which Dr. Peters, or failing him some member of the Association, is asked to prepare a paper for the meeting of the Agricultural Congress to be held at Chicago in October. Dr. Howard suggested that Dr Billings be asked through Dr. Peters to comply with Mr. Appleton's request. Unanimously agreed to. Meeting then adjourned.

JOHN M. PARKER, Secretary.

---

The regular monthly meeting of the Massachusetts Veterinary Association was held at 19 Boylston Place, on Wednesday evening, June 28, 1893, at seven-thirty P. M.

There were present Drs. Beckett, Bryden, Burr, Emerson, Howard, Parker, Peters, Simpson and Winchester. Dr Burr in the chair.

The minutes of the last meeting having been read, Dr. Howard moved that they be accepted as read. Seconded by Dr. Winchester. Carried.

*Dr. Peters* then explained that he had destroyed the notes of the March meeting, and that in many cases he had considered it advisable to omit from the minutes certain portions of the discussions, especially such parts as were at all personal in their nature.

*Dr. Howard* then read from Mr. Bolton's report, and showed that he was not responsible for the portion complained of by Dr Bryden, the mistake being Mr. Bolton's, and not his (Dr. Howard's).

*Dr. Bryden* said it had only been his intention to call attention to a mistake in the "Report."

*Dr. Winchester* then moved that the affair be dropped, and that the minutes of the March meeting stand as they are. Seconded by Dr. Howard. Carried.

*Dr. Winchester* then moved that the minutes of the April meeting stand as they are. Seconded by Dr. Bryden. Carried.

*Dr. Burr* then proposed the name of Dr. William Councilman, M. D., as a member; but on referring to the Constitution it was found he was not eligible for membership.

*Dr. Howard* then moved that William T. Councilman, M. D., of Harvard Medical School, be given an invitation to attend the meetings of the Association, and engage in discussions, and that the Secretary be instructed to send a notice of each meeting. Seconded by Dr. Winchester. Carried unanimously.

*New Business.*—The Secretary then called attention to lapsing of Amendment No. 1 to Article II of Constitution.

*Dr. Winchester* wanted to know what benefits have resulted from the Amendment.

*Dr. Peters* showed that there had been a large increase in the membership.

*Dr Howard* thinks get more members, because many new members do not care to read papers. After considerable discussion, *Dr. Bryden* gave notice that at the next meeting he will move that Article II, Section A, be so amended that said section is rendered inoperative for a period of two years from April, 1893.

*Dr. Winchester* then read open letter from Harvard Veterinary School. He thinks such advertising belittles the profession.

*Dr. Howard* would like to see the subject of Ethics thrashed out. Let those who do not believe in Ethics stand up and defend their position; but if we have a code of Ethics, let us live up to it. It was then arranged that at next (September) meeting the subject of Ethics be thoroughly discussed.

*Dr. Bryden* then read an instructive paper on "Hoof Culture,"* after which an interesting discussion ensued. Dr. Howard thought that there were a great many points in which the members of this Association thoroughly agree with the essayist. (In a greater number perhaps than even he realized.)

*Dr. Peters* then moved a vote of thanks to essayist. Carried unanimously. The meeting adjourned till further notice.

JOHN M. PARKER, Secretary.

---

The regular monthly meeting of the Massachusetts Veterinary Association was held at 19 Boylston Place on Wednesday evening, September 27th, at seven-thirty o'clock. The President, Dr. Burr, in the chair.

The members present were Drs. William M. Balmer, E. C. Beckett, Thomas Blackwood, William Bryden, Alex. Burr, D. Emerson, E. C. Hadcock, L. H. Howard, W. Labaw, Alex. Marshall, F. H. Osgood, J. M. Parker, J. F. Winchester, Chas. Winslow, and Honorary Member Dr. Stickney.

*Dr. Howard* moved that the minutes be accepted as read; seconded by Dr. Blackwood. Carried.

*Dr. Bryden* then moved that "Article II, Section A, of the Constitution be so amended that said section is rendered inoperative for a period of two years from April, 1893." Seconded by Dr. Blackwood. Carried unanimously.

---

* See October JOURNAL.

The Secretary then read a letter from Dr. Hoskins, Secretary to the U. S. V. M. A., asking that delegates be appointed to attend the Veterinary Congress to be held in Chicago during the third week in October. After some discussion on the subject Dr. Osgood suggested that it would be a good plan to do as the Association has done in the past, and appoint as delegates those who intend going to Chicago.

The President then asked the names of those who intended going, and later appointed Drs. Beckett, Winchester and Emerson to act as delegates.

*Dr. Howard* then moved that the Secretary be instructed to communicate with Dr. Hoskins, and inform him that, in compliance with his request, this Association had appointed as delegates Drs. Winchester, Beckett and Emerson. Seconded.

A letter was then read from Dr. Hinkley, Secretary of N. Y. Veterinary Association, with regard to a meeting of the two Associations this year. After a good deal of discussion the subject was dropped, as most of the members seemed to think it would be a complete failure.

The Chairman then called on Dr. Winslow to open the discussion on Ethics. He was followed by Drs. Howard, Blackwood, Winchester and others.

After a lively discussion on this subject, Dr Howard moved that the discussion be continued at the next meeting. Seconded by Dr. Hadcock. Carried. The meeting was then adjourned till next month.

JOHN M. PARKER, Secretary.

